# BPH treatment: laser for everyone | *Opinion: NO*

**DOI:** 10.1590/S1677-5538.IBJU.2018.02.03

**Published:** 2018

**Authors:** Fernando G. Almeida, Luciano Teixeira Silva

**Affiliations:** 1 Departamento de Disfunções Urinárias, Urologia Feminina e Urodinâmica, Universidade Federal de São Paulo, UNIFESP, Escola Paulista de Medicina, São Paulo, SP, Brasil; 2 Departamento de Urologia, Universidade Federal de São Paulo, UNIFESP, Escola Paulista de Medicina, São Paulo, SP, Brasil

**Keywords:** Lasers, Therapeutics, Prostatic Hyperplasia, Transurethral Resection of Prostate

Benign prostate enlargement (BPE) is a highly prevalent pathology (1). The main consequence of BPE is Bladder Outlet Obstruction (BOO). Patients with BOO may be bothered by voiding lower urinary tract symptoms (LUTS). Those men with BOO and significant LUTS which did not respond to clinical approaches may be candidate to surgical procedures. In patients with prostate volume inferior to 80-100 grams, monopolar transurethral resection of prostate (TURP) has been considered the gold standard for decades. The American Urological Association (AUA) considered TURP as standard treatment for BPH (2) and The European Urological Association considered TURP “the treatment of choice” for prostates sized 30 to 80 cm3 (3).

In the past years, a wide range of innovative transurethral procedures have challenged the supremacy of this standard surgical option (4). These alternative transurethral procedures embrace all laser therapies, encompassing the various types of lasers and modalities of prostatic tissue ablation (enucleation, vaporization, and resection) and bipolar devices permitting bipolar TURP (5-7). Many of the “innovative” techniques at their time, such as trans-rectal high intensity focused ultrasound, visual laser ablation and transurethral needle ablation, claimed good results and did not survive to test of the time (8, 9).

TURP has been shown to be cost-effective, efficient, durable and with well-defined long-term complications and re-treatment rates (10). A large prospective multicenter study, including 10,654 men, who underwent TURP described a mortality rate of 0.10% and the cumulative short-term morbidity rate of 11.1% (11). Complications of TURP include failure to void (4.5% to 5.8%), surgical revision (1.1% to 5.6%), urinary tract infection (3.6% to 4.2%), bleeding which requires transfusions (2.0% to 2.9%) and TUR syndrome (0.8% to 1.4%) (11, 12). By using bipolar TURP, TUR syndrome has been overpassed. The bipolar TURP is performed with saline solution, which has improved safety, allow longer resection time and can reduce TUR syndrome, catheter time and length of hospital stay (13). Furthermore, in skilled hands bipolar TURP can be performed in prostate glands bigger than 80-100 grams.

Many endoscopic technologies have been proposed to replace TURP as the new standard reference. There has been a rise in the use of minimally invasive surgical therapy (14). Emerging laser treatments that deserve consideration in this debate are Holmium laser enucleation of the prostate (HoLEP) and photoselective laser vaporization of the prostate (PVP).

There are some trials comparing HoLEP and TURP (15-18). With mean follow--up range of 1 to 3 years, HoLEP demonstrated similar functional results to TURP when considering International Prostate Symptom Score [IPSS], quality of life score [QOL], and maximum flow rate [Qmax]. However, HoLEP operation time was significantly longer in all trials with almost twice the time of TURP in one trial (15). On the other hand, HoLEP can be used as an alternative to open prostatectomy in large prostates (19). It has been demonstrated that HoLEP presented similar functional results, reduced catheterization time, hospital stay, and less blood loss than open prostatectomy for large prostates treatment in two years follow-up (20). Catheter duration, hospital-stay and blood loss were in favour of HoLEP in two meta-analysis (12, 21). Urgency symptoms were more pronounced after HoLEP compared to TURP in one meta-analysis (5.6 vs. 2.2%) (12). Bladder injury during morcellation and postponed morcellation due to equipment failure are reported complications with HoLEP.

The learning curve with HoLEP is a great challenge. Shah et al. described the learning curve of approximately 50 cases (22). Cost is another important issue, particularly in developing countries. The increase in costs are related to the requirement of specific 100W laser, fibers and morcellator need for HoLEP.

Photoselective laser vaporization of the prostate (PVP) uses 532-nm lasers (80-W potassium-titanyl-phosphate [KTP], GreenLight, AMS, Minnetonka, MN) or 120-W lithium borate (LBO) and GreenLight XPS 180W (GL-XPS) (23). It was initially proposed as an alternative to TURP in anticoagulated patients. As opposed to HoLEP, the learning curve of PVP is shorter (24). One inherent limitation of PVP is the absence of tissue diagnosis.

Horasanli et al. showed that immediate outcomes were significantly better in PVP than TURP with reduced time of postoperative catheterization (3.9±1.2 days and 1.7±0.8 days, P<0.05) and shorter length of stay (4.8±1.2 days versus 2±0.7 days, P<0.05). On the other hand, functional improvement (IPSS, Qmax and post-void residual) was significantly worst in PVP, even with shorter follow-up. Operating room times were also significantly longer for PVP (87 vs. 51 minutes) (25). A meta-analysis showed increased dysuria comparing PVP, M-TURP and B-TURP (8.5% vs. 0.8% vs. 0%) and increased postoperative urinary tract infections comparing PVP, M-TURP and B-TURP (12% vs. 4.1 vs. 2.6%) (12). We observe that dysuria may be a significant problem in some patients submitted to green laser surgery. Such problem is minimized in trials, but it is a very common bothering complain and sometimes may last for over three months.

In a randomized controlled trial comparing PVP and open prostatectomy in large glands (average 93 vs. 96mL), surgical room times were significantly longer for PVP (80 vs. 50 minutes) with similar Qmax and IPSS scores, but inferior QOL score in those patients submitted to PVP at the 18-month follow-up (26). Similarly to HoLEP, cost is an issue for PVP/GreenLight laser. Lasers devices are very expensive and fibers are disposable. There are no other usages for this equipment. A trial published on Indian Journal of Urology in 2009, consider that lasers are unreasonable for treatment of BPH, particularly in developing countries, due to costs, unproven long-term durability, steep learning curve and lack of advantages over TURP (27).

We agree with Ahyai et al. (12) that the individual patient's clinical profile should be carefully assessed to identify the most appropriate transurethral technique to manage BOO. Lasers are not appropriate to all patients. There is not single approach for everyone, but a specific patient for each approach. None of the above mentioned therapies are adequate to everyone. We believe that urologists managing symptomatic BPE should be familiar with all above described techniques to be able to judge the best option for each patient. Thus, the surgical approach should be planned based on patient's performance status, use of anticoagulants, prostate volume, personal expectations and surgeon experience.
